# Sugars induced exfoliation of porous graphitic carbon nitride for efficient hydrogen evolution in photocatalytic water-splitting reaction

**DOI:** 10.1038/s41598-024-52593-4

**Published:** 2024-01-23

**Authors:** Daria Baranowska, Klaudia Zielinkiewicz, Ewa Mijowska, Beata Zielinska

**Affiliations:** 1 Department of Nanomaterials Physicochemistry, Faculty of Chemical Technology and Engineering, West Pomeranian University of Technology in Szczecin, Piastow Ave. 42, 71-065 Szczecin, Poland; 2 Center for Advanced Materials and Manufacturing Process Engineering (CAMMPE), West Pomeranian University of Technology, Szczecin, Poland

**Keywords:** Chemistry, Engineering, Nanoscience and technology

## Abstract

Photocatalytic hydrogen evolution holds great promise for addressing critical energy and environmental challenges, making it an important area in scientific research. One of the most popular photocatalysts is graphitic carbon nitride (gCN), which has emerged as a noteworthy candidate for hydrogen generation through water splitting. However, ongoing research aims to enhance its properties for practical applications. Herein, we introduce a green approach for the fabrication of porous few-layered gCN with surface modifications (such as oxygen doping, carbon deposition, nitrogen defects) with promoted performance in the hydrogen evolution reaction. The fabrication process involves a one-step solvothermal treatment of bulk graphitic carbon nitride (bulk-gCN) in the presence of different sugars (glucose, sucrose, and fructose). Interestingly, the conducted time-dependent process revealed that porous gCN exfoliated in the presence of fructose at 180 °C for 6 h (fructose_6h) exhibits a remarkable 13-fold promotion of photocatalytic hydrogen evolution compared to bulk-gCN. The studied materials were extensively characterized by microscopic and spectroscopic techniques, allowing us to propose a reaction mechanism for hydrogen evolution during water-splitting over fructose_6h. Furthermore, the study highlights the potential of employing a facile and environmentally friendly fructose-assisted solvothermal process to improve the efficiency and stability of catalysts based on graphitic carbon nitride.

## Introduction

Hydrogen is an environmentally friendly fuel with a high energy content (142 MJkg^−1^), and its combustion produces only water without releasing carbon dioxide or particulate matter^1^. Moreover, hydrogen is expected to play a vital role in the future as a widespread fuel source. In recent years, hydrogen production has become a global endeavor, primarily through steam reforming of natural gas, coal, or crude oil, with only a small portion coming from biofuel reforming and catalysis^2^. The primary source of hydrogen (95%) is the thermal catalytic reforming of fossil fuels, particularly hydrocarbons, while biomass utilization is considered a second-generation technology^2^. Nevertheless, the substantial heat requirements for thermal catalytic reforming using organic compounds pose a significant challenge to environmental sustainability. Consequently, there is a strong desire to design and develop catalysts capable of efficient water splitting to produce hydrogen with higher yield and specificity, meeting the demands of various industries^3^. One promising solution to address this challenge involves the development of an advanced photocatalyst, harnessing renewable energy sources like solar power to meet the thermodynamic requirements for the photocatalytic splitting of water to release hydrogen.

To design these photocatalysts, various factors need to be considered, including the availability of constituent elements on Earth, cost-effectiveness, not-toxicity, ease of preparation, handling, recyclability, and compatibility with environmentally friendly solvents. In this context, graphitic carbon nitride (gCN) stands out as an excellent candidate^4^. It is a two-dimensional (2D) conjugated polymer composed of carbon and nitrogen, and it possesses several advantageous properties like affordability, lack of metal content, and resistance to both high temperatures and chemical environments^4^. However, the photocatalytic activity of bulk-gCN toward hydrogen evolution frequently falls short of expectations due to its limited surface area, the brief lifespans of photogenerated charge carriers stemming from the π-π conjugated electronic system, and an inadequate photo-redox potential^5^. Therefore, to explore its catalytic potential, several strategies are commonly applied: (i) top-down liquid-phase exfoliation of the bulk material^6^, (ii) defect engineering^7^, (iii) hetero-atom doping^8,9^, or (iv) heterojunction^10^.

As supported by theoretical studies, sugars can act as intercalants for the exfoliation process when treating graphite through mechanochemical methods. Moreover, sugar crystals are readily available, recyclable, and harmless, containing numerous hydroxyl groups. Among the different carbohydrates tasted by Gonzalez et al.^11^, glucose exhibited the most favorable behavior in terms of exfoliation, resulting in the production of few-layer graphene with a relatively limited amount of defects. Considering the similarities between graphitic carbon nitride and graphite in terms of their layered structure, we have presumed that the hydroxyl groups in sugar molecules interact with gCN in a way similar to the graphite delamination^11^. W. Liu et al.^12^ conducted a study where gCN nanoplates were prepared through co-grinding treatment with fructose and sucrose. These few-layered gCN samples exhibited improved photocatalytic performance of hydrogen evolution and Rhodamine B (RhB) degradation under visible light exposure. The rate of H_2_ evolution reached 103.7 and 66.7 µmolh^−1^ g^−1^ for fructose and sucrose co-grinding treatment, respectively, while bulk-gCN displayed a small photocatalytic H_2_ production rate of 37.7 µmolh^−1^ g^−1^. When graphitic carbon nitride was co-grinded with fructose, approximately 98% of RhB was removed within 120 min of visible light irradiation, whereas only 17% of RhB decomposed in the presence of bulk-gCN under identical conditions. In another work, W. Liu et al.^13^ proposed a simple approach involving sugar-assisted exfoliation and subsequent rapid thermal treatment of bulk-gCN to prepare a porous few-layered gCN with nitrogen defects. The process involved co-griding exfoliation of gCN nanoplates in the presence of glucose, followed by short sonication. The as-synthesized gCN nanoplates were subjected to rapid thermal treatment at 700 °C for 2 min, resulting in the repolymerization of heptazine rings and enhancing visible-light photocatalytic activities for water splitting and RhB degradation. The pristine bulk-gCN demonstrated a low photocatalytic hydrogen evolution rate of 37.7 µmolh^−1^ g^−1^, while the exfoliation in the presence of glucose into the few-layered 2D increased the rate to 96.7 µmolh^−1^ g^−1^. The material obtained through the exfoliation-annealing treatment achieved a hydrogen evolution rate of 264.3 µmolh^−1^ g^−1^. Additionally, this sample exhibited efficient degradation of RhB (10 mg/L) after 120 min of illumination, whereas only 17% of RhB could be degraded by the pristine bulk-gCN under the same conditions. S. Sun et al.^5^ demonstrated a hydrothermal method using carbonated beverages (such as Coca-Cola, Pepsi-Cola, Sprite, and Fanta) to reform commercial melamine, resulting in the synthesis of mesoporous graphitic carbon nitride. Nanosheets produced from a melamine precursor reformed with Cola-Cola exhibited 15.1 times greater photocatalytic hydrogen evolution efficiency compared to bulk-gCN, leading to an apparent quantum yield of 7.7% at a wavelength of 420 nm. These findings highlight the noteworthy influence of different sugars on the exfoliation and structural alteration of graphitic carbon nitride.

Inspired by the aforementioned discussion regarding sugar-assisted modifications, we reveal a green strategy to exfoliate, dope, and induce defects on the bulk-gCN surface in a one-step solvothermal method by means of different biocompatible sugar molecules, including glucose, sucrose, and fructose. The obtained results reveal that porous gCN exfoliated in the presence of fructose at 180 °C for 6 h (fructose_6h) exhibits a remarkable 13-fold enhancement in photocatalytic hydrogen evolution compared to bulk-gCN. Comprehensively characterization of the studied materials using microscopic and spectroscopic techniques was conducted, leading to the proposal of a mechanism for hydrogen evolution through water-splitting over fructose_6h. Furthermore, the study highlights the potential of employing a facile and environmentally friendly fructose-assisted solvothermal process to enhance both the efficiency and stability of catalysts based on graphitic carbon nitride.

## Material and methods

### Synthesis of bulk graphitic carbon nitride (bulk-gCN)

A quantity of 5 g of melamine powder (Sigma-Aldrich) was placed in a corundum crucible with a cover. The crucible was then heated in the muffle furnace under air conditions, with a ramp rate of 2 °Cmin^−1^, reaching a temperature of 550 °C. The heating process lasted for 4 h.

### Synthesis of graphitic carbon nitride modified with different sugars (glucose, sucrose, and fructose)

In the subsequent step, 40 mg of various sugars (glucose, sucrose, and fructose sourced from Sigma-Aldrich) were dissolved in a solution of 60 mL of distilled water and ethanol, mixed in a 1:1 volume ratio. Next, 400 mg of bulk-gCN was added to the prepared solution. The mixture was subjected to vigorous stirring for 0.5 h, followed by 0.5 h of sonication. Afterward, the resulting suspension was transferred into a 100 mL Teflon-lined autoclave, where it was maintained at a temperature of 180 °C for 6 h. After cooling down the suspension was centrifuged and washed three times with distilled water and ethanol, followed by drying at 60 °C overnight. The samples were labeled using “sugar_6h”, where “sugar” refers to the type of sugar (e.g., glucose, sucrose, or fructose). For instance, “fructose_6h” signifies that bulk graphitic carbon nitride (bulk-gCN) was modified with fructose, and the reaction duration was 6 h. Moreover, the influence of the solvothermal process time in the presence of fructose on the photocatalytic activity toward hydrogen evolution from water splitting is fully described in *Supplementary Material*. Additionally, bulk graphitic carbon nitride was treated in a solvothermal process but without sugars for comparative study.

### Characterization

The morphology of the studied samples was determined using the Scanning Electron Microscope (SEM) model Vega3 Tescan and the Transmission Electron Microscope (TEM) model Spectra 300, Thermo Fisher Scientific. For Atomic Force Microscope (AFM) analysis, a Nanoscope V Multimode 8 instrument was employed. X-ray Powder Diffraction (XRD) patterns of studied materials were recorded on the Aeris instrument by Malvern Panalytical, utilizing CuKα radiation. The specific surface area was determined by an N_2_ adsorption/desorption isotherm analysis using a Micromeritics ASAP 2460 apparatus. The thermogravimetric tests were performed in air conditions with a temperature range from room temperature to 900 °C with a heating rate of 10 °Cmin^−1^ (DTA-Q600 SDT). The Attenuated Total Reflectance (ATR) method for Fourier Transform Infrared Reflection (FTIR) spectra was acquired using a Nicolet iS5 spectrometer. X-ray Photoelectron Spectroscopy (XPS) measurements were carried out using a Prevac system with a Scienta SES 2002 electron energy analyzer, employing MgKα radiation. Optical absorption spectra of samples were measured using a UV–vis spectrophotometer JASCO V-770, operating in diffuse reflectance mode (DRS) with BaSO_4_ as a white standard. Photoluminescence (PL) spectroscopy was performed on a Hitachi F-7000 instrument with a 380 nm excitation wavelength, while the studied materials were suspended in ethylene glycol. Electrochemical measurements, such as Chronoamperometry (CA) and Electrochemical Impedance Spectroscopy (EIS) were conducted using a three-electrode test cell combined with an Autolab PGSTAT302N instrument. CA tests were performed at 0.5 V vs. SCE with 426 nm excitation, while EIS tests were conducted at 0.15 V vs. SCE in the dark. The counter electrode consisted of a platinum plate, the reference electrode was a saturated calomel electrode (SCE), and 0.1 M Na_2_SO_4_ solution was used as the electrolyte. The working electrode was prepared by dispersion of 2 mg of each catalyst in 1 mL of EtOH:H_2_O with 25 µL of 5 wt% Nafion solution (Sigma Aldrich) by sonication (in a volume ratio of 1:3) and adding). A 50 µL portion of the resulting suspension was deposited onto the fluorine-doped tin oxide (FTO) coated glass (Sigma Aldrich). In the case of studies comparing catalysts before and after photocatalytic reaction, the working electrodes were fabricated using 1 mg of active material.

### Photocatalytic hydrogen evolution

The photocatalytic experiments were conducted in a three-neck Pyrex glass reactor under an Ar atmosphere. Initially, 10 mg of photocatalyst powder was dispersed in 20 mL of water, supplemented with 4 mL of lactic acid (Sigma-Aldrich) as a sacrificial agent. Before each experiment, the system was sealed and purged with Ar gas for 0.5 h. Subsequently, the reactor was illuminated using a 150 W Xe lamp equipped with a 400 nm filter. The gas-phase composition was analyzed using a gas chromatograph (Young Lin 6500), equipped with a 5 Å molecular sieve capillary column (Merck) and TCD (thermal conductivity detector). The volume of the injected gaseous sample was 100 µL. The content of hydrogen in the gaseous phase in subsequent measurements was calculated based on the calibration curve. The stability and recyclability of the most active catalyst were conducted by performing nine consequent cycles of the experiments.

## Results and discussion

**Figure 1:**
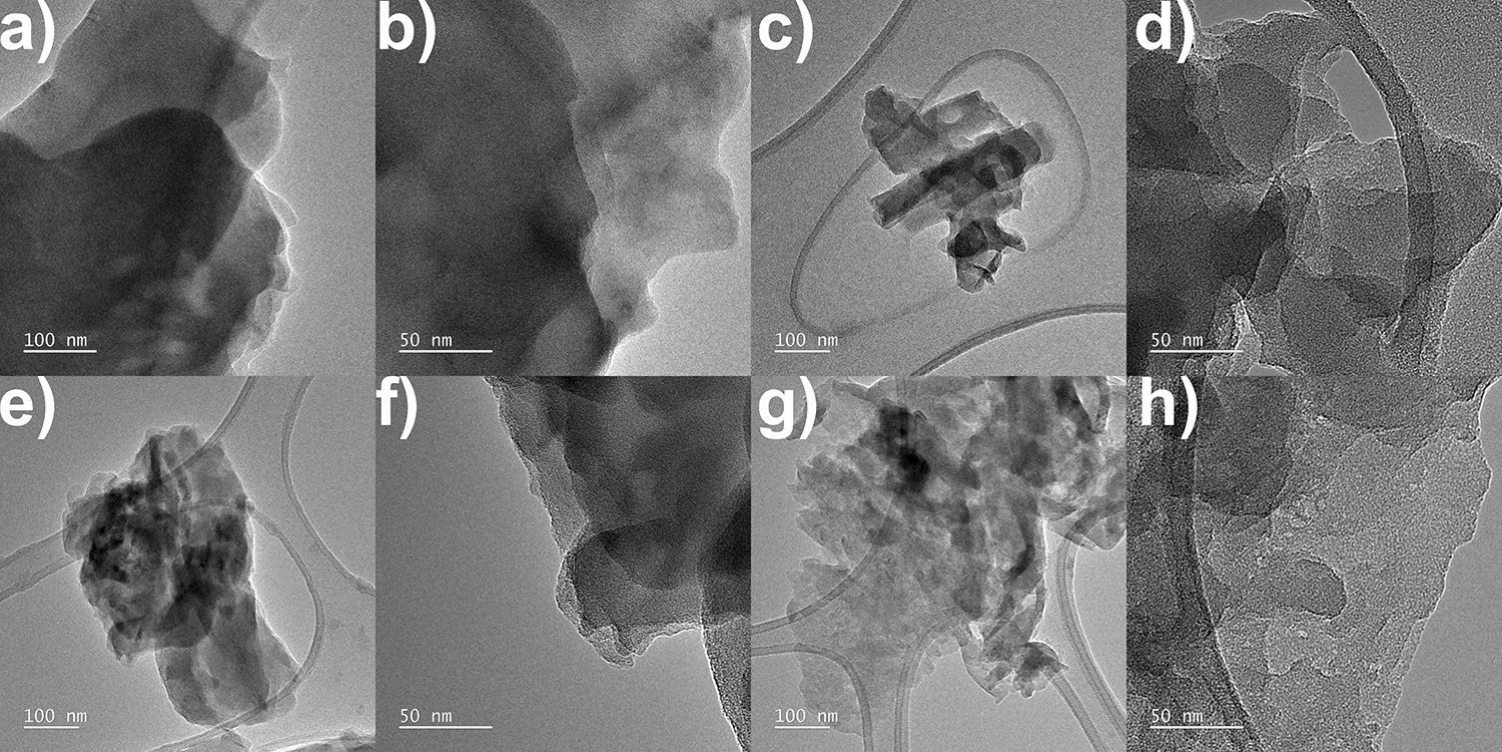
TEM images of (**a**,**b**) bulk-gCN, (**c**,**d**) glucose_6h, (**e**,**f**) sucrose_6h, and (**g**,**h**) fructose_6h.

SEM and TEM images of bulk-gCN, glucose_6h, sucrose_6h, and fructose_6h are displayed in Fig. S1 and Fig. 1, respectively. The bulk-gCN (Fig. S1a & Fig. 1ab) reveals a flat, two-dimensional (2D) plate-like structure, which is a result of strong Coulombic interactions between its carbon–nitrogen units (π–π stacking)^7^. Moreover, it exhibits a distinct composition of smooth-surfaced multilayered nanosheets^14^. The interconnected networks of carbon and nitride atoms in the bulk-gCN appear disorderly over long distances, indicating its amorphous nature^8^. In all sugar-assisted treated samples typical 2D and platelet-like morphology is preserved (Fig. S1b–d)^15^. Interestingly, the effect of the exfoliation process conducted in the presence of various sugars has been revealed. Specifically, the glucose-assisted and fructose-assisted exfoliation processes (glucose_6h—Fig. 1c,d and fructose_6h—Fig. 1g,h) successfully resulted in a more transparent (thinner) layered structure. In contrast, the sucrose-assisted process (sucrose_6h—Fig. 1e,f) did not reduce the thickness of graphitic carbon nitride. The exfoliation process can be explained by the use of sugar-based solvents, which not only increase the lattice spacing between the layers but also weaken the π–π interactions between adjacent layers, resulting in the expansion and exfoliation of bulk-gCN^16^. This occurrence can be explained by the utilization of a high shear mixing in a liquid media (a water–ethanol solution), which generates shear forces that induce vertical expansion and exfoliation of the layers^6^. Moreover, the TEM image of fructose_6h (Fig. 1h) exhibits a highly porous structure, suggesting increased surface area compared to flat bulk-gCN and other sugar-assisted solvothermal treatments. These disparities in thickness and lateral size of sugar-assisted solvothermal exfoliation of bulk-gCN were further confirmed through Atomic Force Microscope (AFM) analyses.

**Figure 2:**
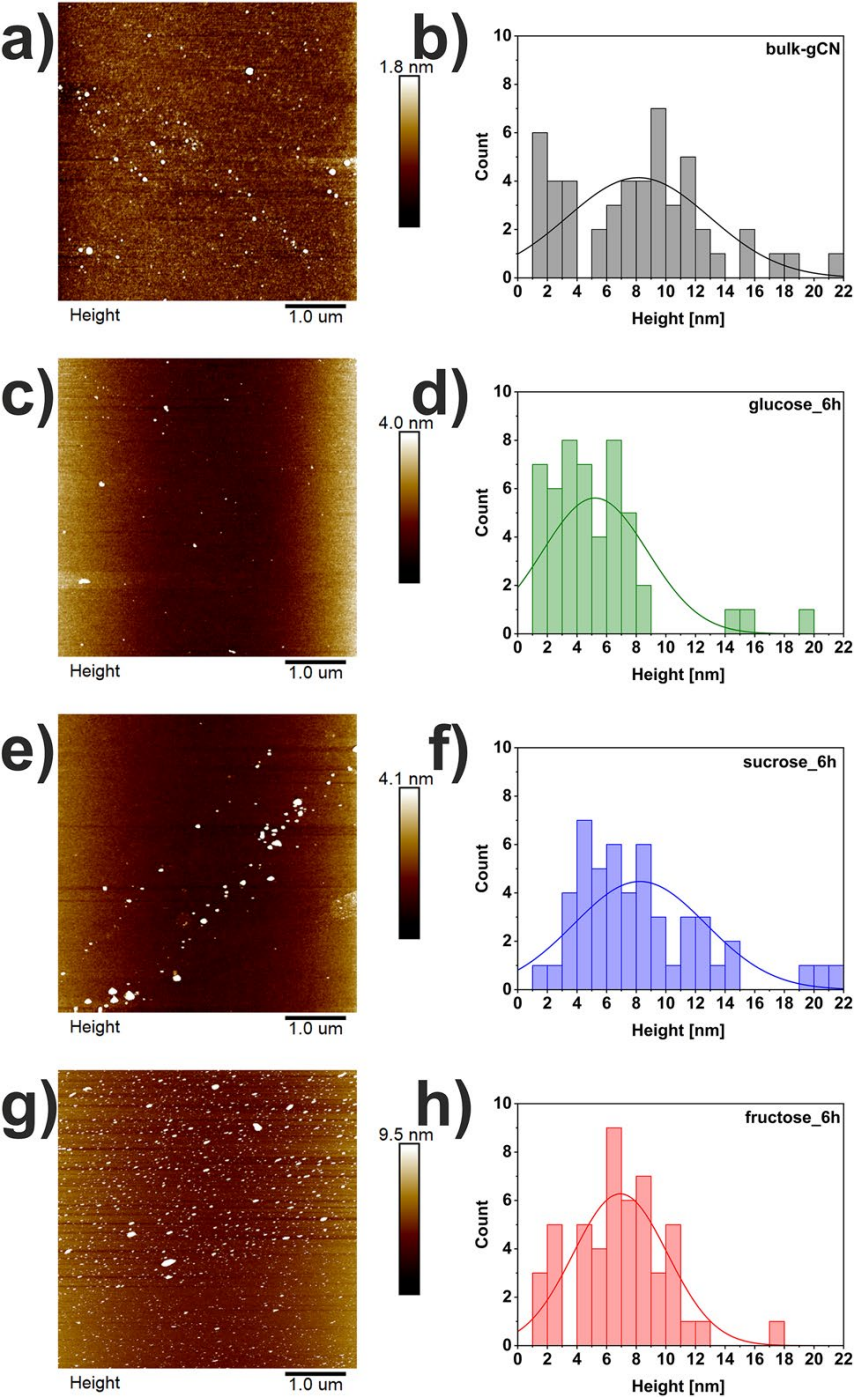
AFM images with corresponding histograms of (**a**,**b**) bulk-gCN, (**c**,**d**) glucose_6h, (**e**,**f**) sucrose_6h, and (**g**,**h**) fructose_6h.

AFM images along with corresponding histograms of bulk-gCN, glucose_6h, sucrose_6h, and fructose_6h are presented in Fig. 2. Additionally, Table S1 lists various parameters, including lateral size, thickness, and the number of layers of studied materials. In detail, for bulk-gCN (Fig. 2a,b), the mean thickness and lateral size are 8.15 nm and 73.0 nm, respectively. Considering the chemical structure of sugars, the abundance of hydroxy groups in their structure is expected to provide an ability to exfoliate multilayered nanomaterials^17^. Following the glucose-assisted process, all aforementioned parameters were reduced. The mean thickness of glucose_6h (Fig. 2c,d) is 5.22 nm, with a mean lateral size of 48.1 nm. The resulting glucose_6h is composed of approximately 16 atomic layers, while the pristine bulk-gCN is composed of 25 atomic layers^18^. The solvothermal treatment involving sucrose (sucrose_6h, Fig. 2e,f) did not lead to any changes in these parameters compared to bulk-gCN, indicating that sucrose alone is insufficient for the exfoliation of graphitic carbon nitride. Similarly to glucose_6h, fructose_6h also exhibited reduced values for the studied parameters. The mean thickness and lateral size of fructose_6h (Fig. 2g,h) are 6.93 nm and 33.5 nm, respectively. The AFM data align with the TEM images (Fig. 1), affirming that sugars, particularly monosaccharides like glucose and fructose, can serve as effective exfoliating agents for graphitic carbon nitride. Although fructose has a chemical structure different from glucose, it can still aid in the exfoliation of bulk-gCN, yet with lower efficiency, attributed to the discriminative ketone group in fructose compared to the aldehyde group in glucose^17^. Monosaccharides will form cyclic structures, leading to various functional groups (aldehyde or a ketone) and different numbers of carbon atoms^17^. This results in different-membered heterocyclic skeletons and a diverse distribution of hydroxyl groups in the ring, which are likely crucial factors influencing the exfoliation efficiency^17^.

The diffractograms of bulk-gCN, glucose_6h, sucrose_6h, and fructose_6h are shown in Fig. S2a. Moreover, the XRD patterns of pristine sugars (glucose, sucrose, and fructose) are presented in Fig. S3a. In detail, bulk-gCN demonstrates two distinct peaks at 12.79 and 27.50°, corresponding to (100) and (002) crystal planes, respectively (JCPDS: 01-087-1526)^19^. The peak at around 13° is attributed to the presence of the tri-s-triazine ring structure in graphitic carbon nitride, while the peak at around 27° signifies the stacking of graphitic layers along the c-axis within the conjugated planes^19^. The diffractograms of graphitic carbon nitride modified with different sugars reveal similar diffraction peaks at (100) and (002) as observed in bulk-gCN, indicating that the primary chemical structure of the graphitic carbon nitride structure remains intact. Thus, sugar-assisted modification of graphitic carbon nitride did not affect the planar structure of the initial graphitic carbon nitride^11^. Notably, the intensities of the diffraction peaks increase with sugar-assisted modifications, indicating an enhanced conjugated aromatic system and improved crystallinity^13^. The improved crystallinity can be attributed to the removal of less-ordered domains in graphitic carbon nitride during the exfoliation process^19^.

The Thermogravimetric Analysis (TGA) results of bulk-gCN, glucose_6h, sucrose_6h, and fructose_6h are displayed in Fig. S2b. The *Supplementary Material* (Fig. S3b) contains TGA curves of pure sugars (glucose, sucrose, and fructose). The thermal analysis curves reveal subtle differences in the thermal behavior of the studied samples. Specifically, the primary weight loss observed in all samples at approximately 700 °C can be attributed to the decomposition and condensation of the graphitic carbon nitride structure^20^. Notably, the TGA curve of fructose_6h exhibits the highest degree of interaction with graphitic carbon nitride compared to other sugar-assisted modifications of gCN, which can be observed by the fastest loss of ketone group from the surface of functionalized gCN. This can be attributed to the lower melting point of fructose (103 °C) compared to other sugars: glucose (148 °C) and sucrose (179 °C)^21^. These results suggest that the modification of graphitic carbon nitride with fructose at 180 °C is the most effective approach among the tested sugars. This modification results in a highly functionalized gCN surface, which can boost its photocatalytic activity.

**Figure 3:**
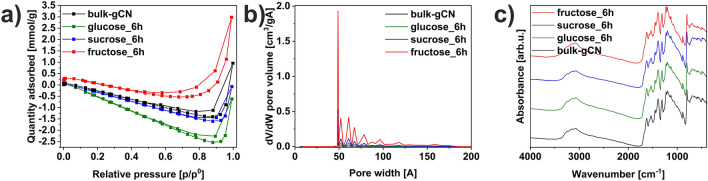
(**a**) nitrogen adsorption–desorption isotherms, (**b**) pore size distribution, (**c**) FTIR-ATR spectra of bulk-gCN, glucose_6h, sucrose_6h, and fructose_6h.

The nitrogen adsorption/desorption isotherms of bulk-gCN, glucose_6h, sucrose_6h, and fructose_6h are presented in Fig. 3a. The graphitic carbon nitride modified with different sugars exhibited an isotherm characterized by a type IV shape, along with an H4-shaped hysteresis loop. The N_2_ adsorption isotherm of all studied materials showed the highest adsorption at high relative pressures, corresponding to the presence of numerous mesopores^5^. The adsorption of N_2_ at low pressure is related to the filling of micropores, suggesting the existence of a weak microporosity^8^. The specific surface area was determined using Brunauer–Emmett–Teller (BET) analysis and calculated to be 8.74, 3.54, 1.12, and 12.47 m^2^g^-1^ for bulk-gCN, glucose_6h, sucrose_6h, and fructose_6h, respectively. These results indicate that only the fructose-assisted solvothermal modification of graphitic carbon nitride (fructose_6h) improved the specific surface area by a factor of 1.43 compared to pristine bulk-gCN. A similar dependence was observed for the total pore volume (Fig. 3b). The total pore volume was measured as 3.08, 1.42, 0.37, and 3.88 mm^3^g^−1^ for bulk-gCN, glucose_6h, sucrose_6h, and fructose_6h, respectively. Similarly, only fructose_6h has a higher pore volume, while the other sugars present a lower value of this factor than bulk-gCN. Importantly, the high specific surface area and large pore volume can significantly enhance photocatalytic reaction kinetics by facilitating reactant molecules' movement^5^. The increased surface, along with the reduction in the thickness of graphitic carbon nitride (Fig. 2 & Table S1), promotes the generation of more reactive sites for the adsorption of reactants and facilities charge transfer to the active sites on the surface, thereby benefiting the photocatalytic applications.

The FTIR-ATR spectra of bulk-gCN, glucose_6h, sucrose_6h, and fructose_6h are presented in Fig. 3c. The *Supplementary Material* includes absorption spectra of (Fig. S3c) pristine sugars (glucose, sucrose, and fructose). The absorption peaks observed in the spectra of all studied materials were similar, indicating that the chemical structure of graphitic carbon nitride remained intact. The presence of these similar characteristic peaks in the analyzed samples confirmed that the 2D hexagonal framework of graphitic carbon nitride remained unchanged. Thus, the results are in good agreement with XRD results (Fig. S2a). This preservation is crucial for maintaining the π-delocalized electronic structure, which is responsible for generating and transferring charge carriers for the subsequent redox reactions^13^. Specifically, a distinct peak at 803 cm^−1^ was identified as the respiratory mode of the heptazine ring (s-triazine units)^22^, indicating that the structure of graphitic carbon nitride was maintained. The fingerprint region located between 1100 and 1700 cm^−1^ exhibited stretching vibration modes specific to aromatic C=N/C–N heterocycles (aromatic carbon and nitrogen heterocycles)^23^, which are characteristic of s-triazine units^24^. Broad peaks between 3000 and 3400 cm^−1^, arising from N–H/O–H stretching vibrations, were observed, suggesting the hydrogenation of nitrogen atoms in the nanosheets^25^.

**Figure 4:**
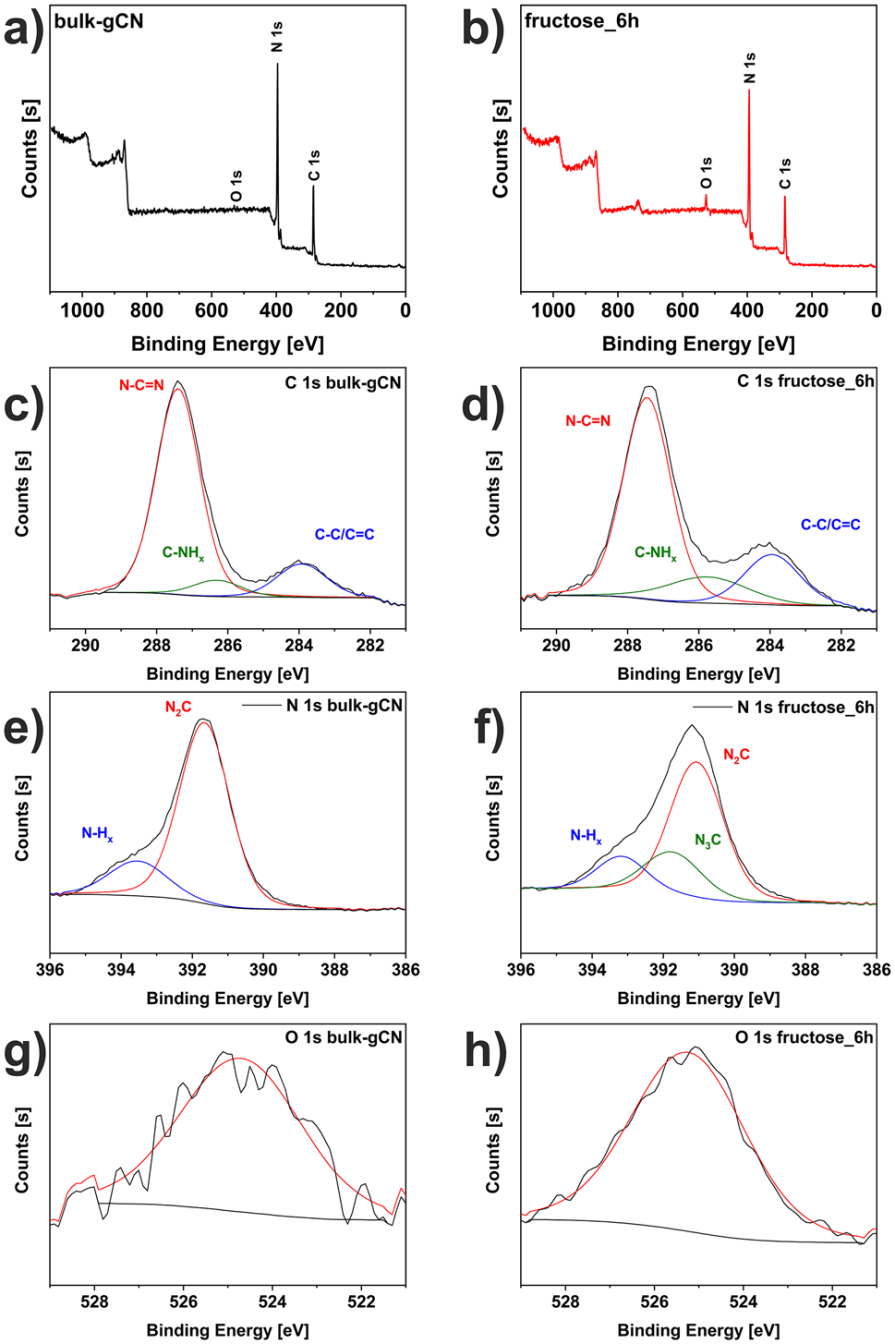
XPS survey spectra of **(a**) bulk-gCN, (**b**) fructose_6h. C 1 s spectra of (**c**) bulk-gCN, (**d**) fructose_6h. N 1 s spectra of (**e**) bulk-gCN, (**f**) fructose_6h. O 1 s spectra of (**g**) bulk-gCN, (**h**) fructose_6h.

X-ray Photoelectron Spectroscopy (XPS) was utilized to analyze the composition and chemical states of the unmodified sample (bulk-gCN) and material with the highest photocatalytic activity toward hydrogen generation (fructose_6h) (Fig. 4). The survey spectra of bulk-gCN *(*Fig. 4a) and fructose_6h (Fig. 4b) exhibit distinct peaks of C 1 s, N 1 s, and O 1 s. The atomic concentrations of these elements are listed in Table 1. The result indicated that in fructose_6h the amount of carbon and oxygen increased, from 41.04 to 43.02 at% and from 0.97 to 3.71 at%, respectively, while the amount of nitrogen decreased (from 57.99 to 53.77 at%). The increased carbon content in fructose_6h provides evidence for carbon formation and deposition on the graphitic carbon nitride surface, which is a result of the carbonization of fructose during the solvothermal process. Carbon deposition onto the surface of the graphitic carbon nitride induces additional porosity, effectively suppressing the recombination rates, and improving the transfer and separation of photogenerated carriers on its surface^26^. On the other hand, the introduction of oxygen atoms into the graphitic carbon nitride framework is beneficial for broadening the light absorption range and suppressing charge recombination, thus enhancing photoactivity^13^. Indeed, the insertion of oxygen-containing functional groups by solvothermal process greatly favors the material dispersion in aqueous solution and further enhances its interfacial coupling and redox activity by an effective electron doping with extra-electron redistribution in the nearest C-atoms and delocalization among the π-bonds^27^. Additionally, quantitative analyses reveal a slightly increased C/N molar ratio in fructose_6h (0.80) compared to bulk-gCN (0.71). This increase in the C/N ratio can indicate the presence of a nitrogen-vacancy defect^13^. The detailed analysis of the individual components was calculated using the peak-fitting procedure to C 1 s and N 1 s spectra of studied samples and presented in Table 1. In detail, Fig. 4c,d presents the C 1 s spectrum, where the most intense peak at 287.4 eV is attributed to the N–C = N sp^2^ carbon–nitrogen bonds present in the aromatic ring system, specifically the s-triazine unit^4^. This peak is considered the primary carbon species in graphitic carbon nitride^28^. The less intense peak which is centered at 283.9 eV corresponds to the graphitic C–C/C=C bonds^4^. The weak peak centered at 286.3 eV is assigned to 3° nitrogen in the C-NH_x_ group, where nitrogen is trigonally bonded to sp^2^ carbon atoms in the C-N network^4^. Figure 4e,f illustrates the N 1 s spectrum, which displays three peaks at binding energies of 391.7, 391.8, and 393.6 eV. The main signals at 391.7 eV can be assigned to sp^2^ pyridinic N involved in triazine rings (N_2_C)^4,5^, indicating the presence of triazine rings^28^. The peak at 391.8 eV corresponds to sp^3^ hybridized tertiary nitrogen atoms of N_3_C^4,5^. The peak at 393.6 eV corresponds to pyrrolic or pyridine N groups (N-H_x_)^4,5^, suggesting the exitance of the defects on the catalyst surface^28^ or the presence of amino functional groups^13^. It was noticed that the fructose-assisted modification of graphitic carbon nitride causes the increased concentration of the following bonding: C-NH_x_ (from 7.01 to 14.65 at%), C–C/C=C (from 14.59 to 19.16 at%) and N_3_C (from 0.00 to 21.48 at%). Furthermore, within the process of exfoliation of bulk-gCN, surface modifications, including oxygen doping, carbon deposition, or the introduction of nitrogen defects, result in a carefully designed catalyst derived from graphitic carbon nitride specifically for photocatalytic hydrogen evolution.

**Table 1:** Carbon, nitrogen, and oxygen atomic concentrations with chemical composition calculated from the peak-fitting procedure applied to the C 1 s and N 1 s spectra of bulk-gCN and fructose_6h.

Sample	C (at%)	N (at%)	O (at%)	N–C=N (at%)	C–NH_x_ (at%)	C–C/C=C (at%)	N–H_x_ (at%)	N_3_C (at%)	N_2_C (at%)
bulk-gCN	41.04	57.99	0.97	78.39	7.01	14.59	18.12	–	81.88
fructose_6h	43.02	53.77	3.71	66.19	14.65	19.16	14.81	21.48	63.71

**Figure 5:**
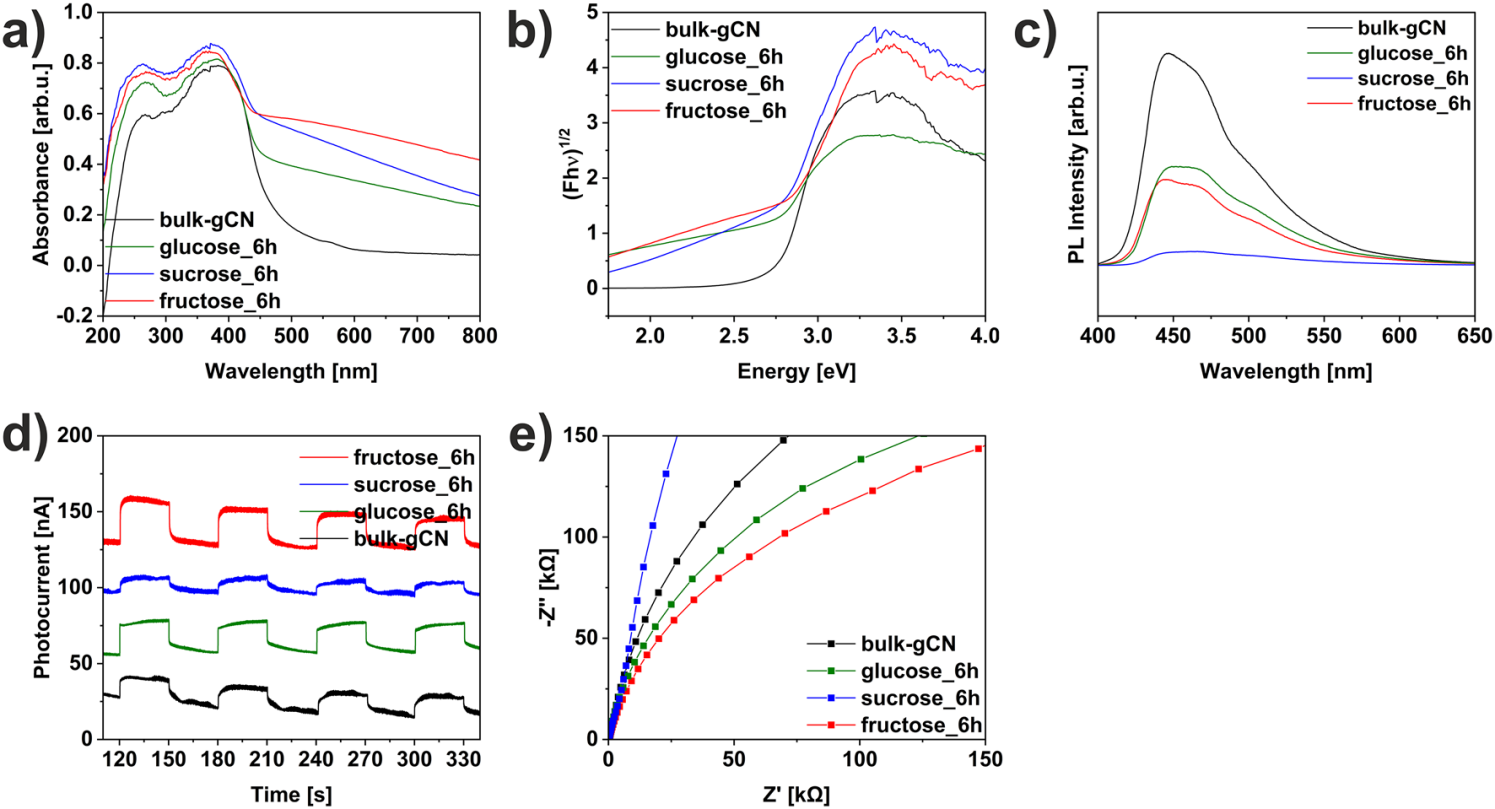
(**a**) DR/UV–vis spectra, (**b**) Tauc plot, (**c**) Photoluminescence emission spectra, (**d**) Chronoamperometry, (**e**) Electrochemical Impedance Spectroscopy of graphitic carbon nitride modified with different sugars (glucose, sucrose, fructose).

The UV–visible absorption spectra of bulk-gCN, glucose_6h, sucrose_6h, and fructose_6h are demonstrated in Fig. 5a. In detail, the fundamental absorption edges were observed around 470 nm, indicating intrinsic semiconductor-like absorption in the blue region of the visible light spectrum^28^. Additionally, the UV–vis absorption of all studied materials displayed two intrinsic peaks at 263 and 371 nm. The broad band at 263 nm was attributed to the π–π* electron transitions of the graphitic carbon nitride’s carbon core sp^2^ aromatic structure (C=C conjugate system)^14^. Another band at 371 nm may be linked to the surface states, which result from the n–π* electron transitions of the C=O and C–NH_2_ groups on the surface of all studied materials^14^. The modification of graphitic carbon nitride with different sugars influenced the band structure and affected the position of the band gap^14^. This was evident from the observed hyperchromatic shift, caused by the formation of additional carbon on the graphitic carbon nitride surface resulting from sugar carbonization during the solvothermal process. Additionally, the light absorbance of sugar-modified graphitic carbon nitride in the region of 500–800 nm was noticeably enhanced, primarily due to the introduction of interband defect states caused by the nitrogen vacancies^13^, confirmed by the XPS technique (Fig. 4 & Table 1). The sugar-assisted solvothermal modification of graphitic carbon nitride led to a slight hypsochromic shift, resulting in a blue shift of the band gap. The energy band gaps (Fig. 5b) were determined as 2.75, 2.79, 2.81, and 2.89 eV for bulk-gCN, glucose_6h, sucrose_6h, and fructose_6h, respectively. Analyses of the Tauc plot revealed a progressively broadened bandgap, ranging from 2.75 eV in bulk-gCN to 2.89 eV in fructose_6h. The increased bandgap of the sugar-modified graphitic carbon nitride resulted from the reduction in the thickness (Fig. 2 & Table S1), which can be attributed to the quantum confinement effect^29,30^. Consequently, the broadening of the band gaps is likely a result of the quantum confinement effect induced by the successful exfoliation of graphitic carbon nitride with different sugars^13^. This phenomenon accelerates the migration and separation of the photogenerated carriers^31^, thus promoted photoactivity is expected.

The investigation of photoluminescence (PL) emission spectra of bulk-gCN, glucose_6h, sucrose_6h, and fructose_6h was conducted to analyze the recombination probabilities of photogenerated carriers*. *Figure 5c shows the PL spectra of the studied samples excited at room temperature with 380 nm wavelength. Pristine bulk-gCN exhibited a strong intrinsic fluorescence emission peak at 445 nm, which corresponds to the rapid recombination of photogenerated carriers in the band-to-band transition^14^. The emission is attributed to the n–π* transitions^32^. Furthermore, two peaks centered at 466 and 500 nm are evident, indicating the presence of two recombination centers^33^. This can be attributed to the existence of multiple active energy levels contributing to the process of photoemission^34^. Specifically, the peak centered at 466 nm is associated with the transition from π* to π, while the peak centered at 500 nm corresponds to the transition from π* to the conduction band with long-pair electrons^35^. The fundamental structure of graphitic carbon nitride consists of tri-s-triazine ring units connected by nitrogen atoms, forming a conjugated polymeric network^36^. The optical properties of carbon-based materials with the disorder are governed by the π and π* antibonding states within the UV–visible energy range^36^. The luminescence behavior of carbon nitride materials is influenced by the size of the sp^2^ C–N structure, and the presence of long-pair electrons associated with the nitride^36^. Furthermore, the formation of the long-pair state arises from the non-hybridization of the long-pair electrons with carbon, leading to the sp^2^ C–N π valance band^36^. However, noticeable reductions in the PL intensity and enhanced PL quenching of the graphitic carbon nitride modified with different sugars were observed. This indicates the effective inhibition of electron–hole pair recombination on the surface of graphitic carbon nitride^5^. The observed PL quenching confirms a suppressed recombination rate of the photo-induced charge carriers^37^. Furthermore, the PL spectra also reveal the presence of a quantum confinement effect, as the sugar-modified materials exhibit a blue shift compared to bulk-gCN, consistent with the absorption spectrum (Fig. 5a). The reduced intensity in the PL spectrum reflects the relocalization of electrons on the surface terminal sites, which is beneficial for enhancing photocatalytic performance^13^. The quenching of the emission intensity suggests that a portion of carbon nitride excitations occurs through nonradiative paths, involving charge transfer of electrons and holes to newly localized states, which are attributed to the surface modifications of graphitic carbon nitride during a single-step solvothermal process utilizing different sugars^38^.

The separation efficiency of photogenerated carriers of bulk-gCN, glucose_6h, sucrose_6h, and fructose_6h was investigated using Chronoamperometry (CA) tests under 426 nm excitation wavelength, as depicted in Fig. 5d. The results demonstrate that graphitic carbon nitride modified with different sugars exhibits reproducible photoresponses during multiple on/off cycles under visible light, indicating effective utilization of visible light for photocatalysis. Among all studied samples, fructose_6h shows the highest photocurrent response, indicating significantly enhanced separation efficiency of photo-induced electrons and holes^5^. This finding further confirms the improved separation and migration efficiency of photogenerated charges^14^ and highlights the superior charge separation capability of fructose_6h^13^. Electrochemical Impedance Spectroscopy (EIS) Nyquist plots of the studied materials are illustrated in Fig. 5e. Notably, the Nyquist plot of fructose_6h exhibits the smallest semicircle diameter compared to the other samples, indicating the highest efficiency in separating photogenerated electron–hole pairs and facilitating charge transfer at the solid/liquid interface^14^. This observation suggests that the fructose modification of graphitic carbon nitride results in lower resistivity^14^. The decrease in semicircle diameter indicates improved electronic conductivity in the non-photoexcited state and accelerated migration of charge carriers^39^. The smaller semicircular diameter in the Nyquist plots of graphitic carbon nitride modified with different sugars compared to bulk-gCN can be attributed to higher conductivity, thus reducing the resistance to photoelectron transfer^7^. Among the studied samples, fructose_6h demonstrates advantageous charge behavior, which can be attributed to the introduction of nitrogen defects and the presence of a porous few-layered structure, both of which promote charge separation and transfer^13^. Moreover, surface modifications, such as oxygen doping, carbon deposition, and nitrogen vacancies are beneficial in promoting the surface conductivity of catalysts, which would enhance the prosperity of transferring electrons and charged particles^40^. The results obtained from Electrochemical Impedance Spectroscopy are in full agreement with Chronoamperometry analyses. Therefore, based on the optical and electrochemical properties of the studied materials, it can be concluded that an appropriate synthesis pathway involving the modification of graphitic carbon nitride with different sugars can effectively enhance the separation and transfer of photoinduced charges, thereby improving photocatalytic activity^14^.

**Figure 6:**
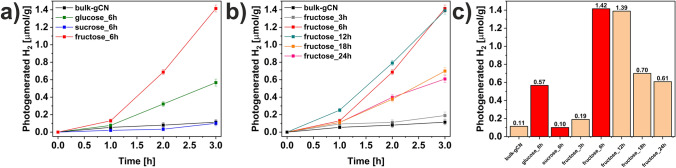
Hydrogen evolution from water splitting catalyzed by (**a**) graphitic carbon nitride modified with different sugars (glucose, sucrose, and fructose), (**b**) graphitic carbon nitride modified with fructose—influence of the solvothermal process time, (**c**) comparison of photogenerated H_2_ after 3 h of photocatalytic reaction.

The impact of surface modifications of graphitic carbon nitride through a facile and environmentally friendly solvothermal exfoliation using various sugars (glucose, sucrose, and fructose) was studied toward photocatalytic hydrogen evolution from water splitting. The results are displayed in Fig. 6. The photocatalytic experiments were conducted under visible light illumination with lactic acid (LA) as a sacrificial agent, without the presence of Pt as a co-catalyst. It should be noted that LA is commonly used as a sacrificial agent in photocatalytic hydrogen production. The pristine bulk-gCN showed minimal hydrogen evolution (0.11 µmolg^−1^) after 3 h of visible light exposure. Interestingly, not all sugar-assisted modifications of graphitic carbon nitride improved its photoefficiency. In the case of sucrose_6h, the hydrogen evolution decreased to 0.10 µmolg^-1^. This decrease in photoactivity of sucrose_6h may be attributed to (i) the smallest surface area, and (ii) the lowest photoresponses (CA) and the highest semicircle diameter (EIS), indicating low mobility of charge carriers on its surface. On the other hand, modifications with monosaccharides, such as glucose and fructose, led to an increase in hydrogen evolution, with glucose_6h and fructose_6h achieving 0.57 and 1.42 µmolg^−1^, respectively, which is an impressive increase in respect to the activity of bulk-gCN (0.11 µmolg^−1^). Due to the highest efficiency of hydrogen evolution over fructose_6h, the optimization of solvothermal process duration (3, 6, 12, 18, 24 h) with fructose was extensively studied. The physiochemical characterization, including XRD, FTIR-ATR, DR/UV–vis with corresponding Tauc plot, PL, CA, and EIS, of fructose-assisted solvothermal process optimization is presented in Supplementary Material (Fig. S4). The reaction durations of 6 and 12 h showed similar high efficiency, resulting in hydrogen evolution up to 1.42 and 1.39 µmolg^-1^ for fructose_6h and fructose_12h, respectively. However, prolonging the reaction time to 24 h led to a decrease of photogenerated hydrogen to 0.61 µmolg^-1^ for fructose_24h. To highlight the significant impact of sugar presence in the fabrication of the studied catalyst, the reference sample of bulk-gCN in a solvothermal process in the absence of sugars was produced. The SEM, TEM, AFM, and XRD analyses with hydrogen evolution from water splitting are shown in Fig. S5. The morphology of reference bulk-gCN seems not to be changed (Fig. S5a–c), otherwise, TEM images present a very thin 2D structure. The mean thickness of reference bulk-gCN (Fig. S5d) is 3.09 nm, suggesting that as-fabricated material is composed of 10 layers, confirming that the solvothermal process (distilled water and ethanol, mixed in a 1:1 volume ratio) is an effective approach to exfoliate graphitic carbon nitride. The diffractogram (Fig. S5e) shows characteristic peaks of graphitic carbon nitride. The hydrogen evolution of reference bulk-gCN (Fig. S5f, gray line) shows a slight increase of H_2_ evolution (0.19 µmolg^−1^) after 3 h of visible light exposure compared to unmodified bulk-gCN (0.11 µmolg^−1^), confirming that the presence of sugars, especially glucose, and fructose, is crucial for the fabrication of catalyst based on graphitic carbon nitride with boosted activity toward photocatalytic hydrogen evolution from water splitting.

**Figure 7:**
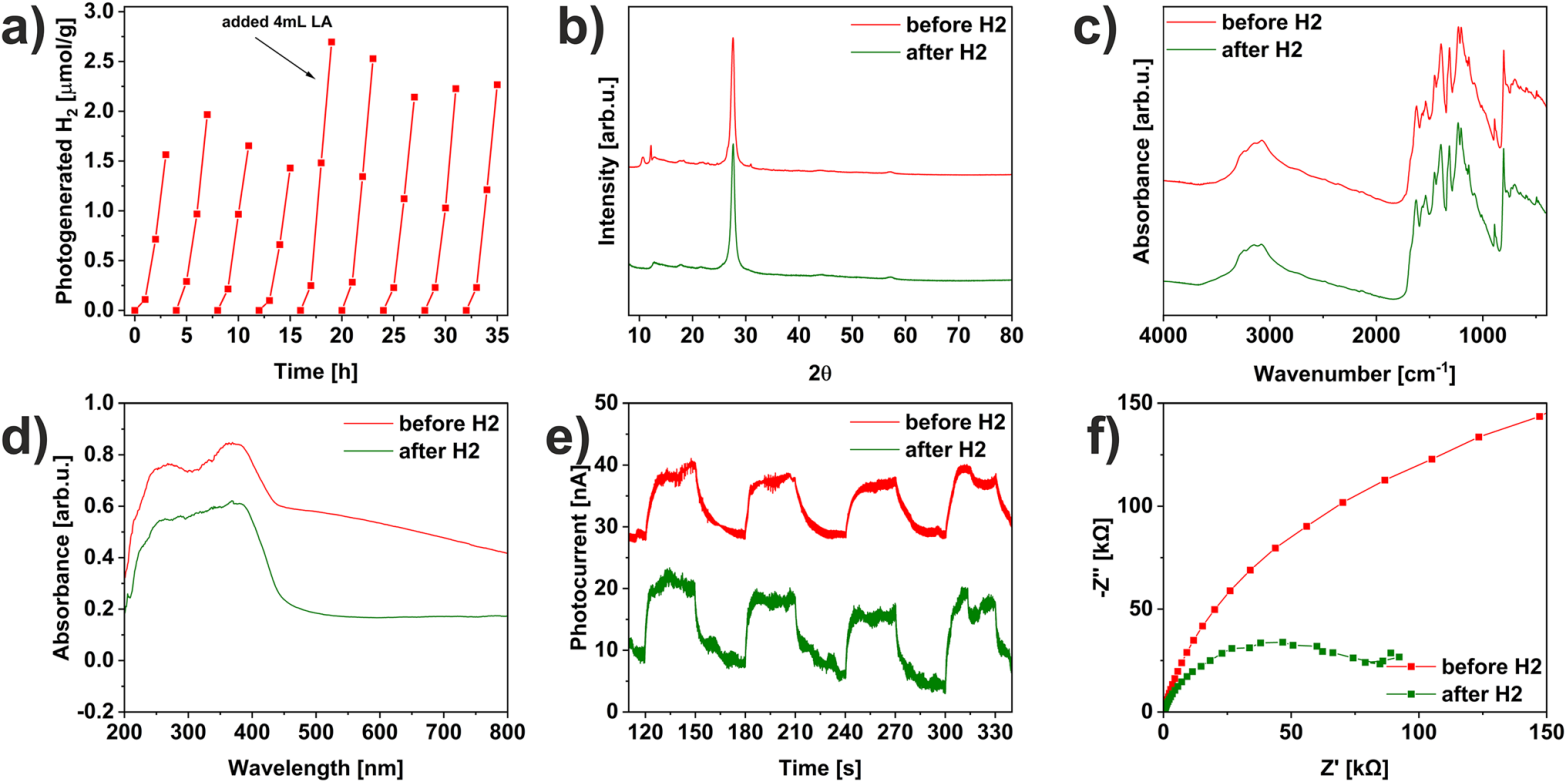
(**a**) Stability test over fructose_6h, comparison of fructose_6h before and after stability test using (**b**) XRD, (**c**) FTIR-ATR, (**d**) DR/UV–vis, (**e**) CA and (**f**) EIS techniques.

Therefore, fructose_6h has been tested in greater detail to determine its stability. It was demonstrated in sustainable photocatalytic H_2_ evolution for up to nine cycles, lasting 35 h (Fig. 7a). The cycle tests conducted under identical reaction conditions reveal that the fructose_6h exhibits enhanced photocatalytic H_2_ generation in the second cycle (1.97 µmolg^−1^), followed by a gradual decrease in photoefficiency. This decrease in photoactivity during prolonged photocatalytic reactions is commonly observed due to the partial consumption of sacrificial agent. To address this issue, in the fifth cycle, an additional portion of LA was added to the reactor, resulting in increased hydrogen evolution (2.69 µmolg^−1^). Following this, a slight decrease was observed but next the stabilization in the efficiency of hydrogen evolution up to 35 h was observed. To investigate the mechanism behind the photocatalytic hydrogen evolution in the presence of fructose_6h, the recycled fructose_6h, after the photocatalytic process was characterized using XRD, FTIR, DRS, CA, and EIS techniques to assess its structural, optical, and electrochemical integrity. The XRD diffractogram (Fig. 7b) of the material after the photocatalytic reaction exhibits less pronounced peaks compared to the material prior to hydrogen evolution, indicating a significant reduction in the planar size and thickness of carbon nitride during the photocatalytic reaction^24,41^. The FTIR-ATR spectra (Fig. 7c) show similar absorption peaks. The DR/UV–vis analysis (Fig. 7d) demonstrates that the absorption edge of the catalyst after the photoreaction is more prominent, with a hypsochromic shift of the absorption edge, indicating a narrowing of the energy band gap from 2.89 to 2.74 eV^42,43^. The electrochemical properties, as presented in Fig. 7e,f, show comparable photoresponses for both materials, suggesting similar mobility of charge carriers, while the resistance of fructose_6h after the H_2_ generation reaction is highly improved.

**Figure 8:**
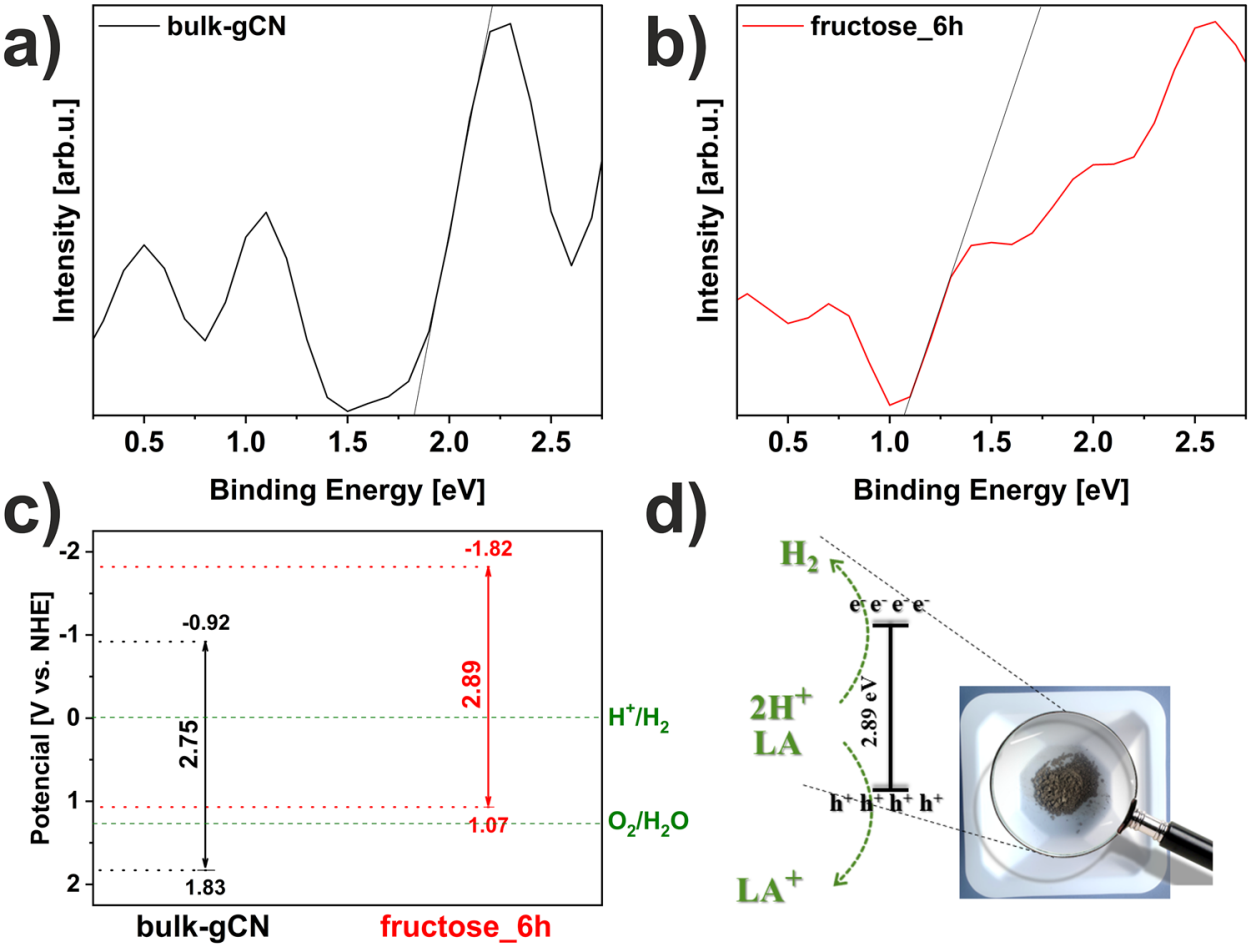
VB XPS spectra of (**a**) bulk-gCN and (**b**) fructose_6h. (**c**) Optical band gaps diagram of studied catalysts. (**d**) Proposed mechanism of hydrogen evolution in photocatalytic water-splitting reaction over fructose_6h.

To assess the quality of the proposed catalyst it is clear that it meets the thermodynamic requirements for the photocatalytic splitting of water to release hydrogen. To elaborate further, the flat band potential values of bulk-gCN and fructose_6h determined through valance band XPS (VB XPS) plots were established to be + 1.83 and + 1.07 V, respectively (Fig. 8ab). The conduction band (CB) potential (E_CB_) values were calculated using the equation E_g_ = E_VB_ – E_CB_^14^. The energy band gaps (E_g_) values of pristine bulk-gCN and fructose_6h were 2.75 and 2.89 eV, respectively (Fig. 5b), while their corresponding E_CB_ values were − 0.92 and − 1.82 V, respectively. The up-shifting in the conduction band (CB) value of fructose_6h compared to bulk-gCN (Fig. 8c) can be explained by the influence of quantum confinement effects, which broaden bandgap values^5^. This enlarged CB indicates that the fructose_6h sample possesses stronger redox capabilities than bulk-gCN, making photo-generated electrons more reductive and enhancing their photo-reduction reactions. Furthermore, nitrogen vacancies in fructose_6h result in additional electrons being redistributed to neighboring carbon atoms and delocalized within the π bonds of the graphitic carbon nitride structure^13^.

A proposed mechanism of hydrogen evolution through photocatalysis over fructose_6h is illustrated in Fig. 8d. Pure bulk-gCN is limited by its large E_g_ and rapid recombination of photogenerated carriers, leading to inefficient photocatalytic performance under visible-light irradiation^14^. However, the fructose-assisted solvothermal process of graphitic carbon nitride enables efficient hydrogen generation from water-splitting^14^. Fructose_6h possesses a mesoporous structure with reduced thickness, providing a high specific surface area to accommodate active sites for the H^+^/LA reactants and facilitating the rapid transport of H^+^/LA through its architecture^14^. Moreover, the structure of fructose_6h also enhances light harvesting through inner light reflection, which contributes to improved photocatalytic activities^14^. Additionally, surface modifications in fructose_6h, such as oxygen doping, carbon deposition, and nitrogen vacancies, can act as separation centers, capturing photogenerated electrons from the CB and promoting faster charge separation^14^. Moreover, a higher degree of crystallinity in fructose_6h is advantageous for the uphill reactions of water splitting due to the reduced presence of recombination centers^14^. Consequently, an impressive enhancement in photocatalytic performance is achieved by combining the tailored crystal, textural, optical, and electronic structures^14^. Furthermore, the presented findings suggest that the process of photocatalytic hydrogen evolution from water splitting is complex, influenced by various distinct factors including morphology, thickness, surface modifications, crystallinity, energy band gap, recombination process, separation and transport of photogenerated charges carriers, resistance of catalyst based on graphitic carbon nitride. All of these elements collectively affect the photocatalytic H_2_ efficiency.

## Conclusions

In summary, we have developed a green approach for the fabrication of porous few-layered graphitic carbon nitride designed for use as a photocatalyst in the hydrogen evolution reaction. The fabrication process involves a one-step solvothermal treatment of bulk graphitic carbon nitride (bulk-gCN) in the presence of various biocompatible sugars such as glucose, sucrose, and fructose. The conducted time-dependent process revealed that porous graphitic carbon nitride exfoliated in the presence of fructose at 180 °C for 6 h (fructose_6h) exhibits a superior performance of photocatalytic hydrogen evolution compared to pristine bulk-gCN. The boosted photoactivity of fructose_6h can be attributed to several factors, including (i) porous 2D structure, (ii) surface modifications, (iii) improved separation and transfer of charge carriers, (iv) suppressed recombination probabilities, (v) enlarged conductive band edge, thereby maximizing the photocatalytic performance through water splitting reaction. Furthermore, the study highlights the potential of employing a facile and environmentally friendly solvothermal process assisted by fructose to improve the efficiency and stability of catalysts based on graphitic carbon nitride. Moreover, this approach can be successfully extended for the green exfoliation of other 2D materials.

### Supplementary Information

Supplementary Information.

## Data Availability

The datasets used and/or analyzed during the current study are available from the corresponding author upon reasonable request.
