# Supplementary material for: Sugars induced exfoliation of porous graphitic carbon nitride for efficient hydrogen evolution in photocatalytic water-splitting reaction
Source: Sci Rep. 2024 Jan 23;14:1998. doi: 10.1038/s41598-024-52593-4 (PMC10805789; doi:10.1038/s41598-024-52593-4)
Supplement: Supplementary file 1 — Supplementary Information. [file 41598_2024_52593_MOESM1_ESM.pdf]

Supplementary Material

Sugars induced exfoliation of porous graphitic carbon nitride for efficient hydrogen evolution in photocatalytic water-splitting reaction

Daria Baranowska*, Klaudia Zielinkiewicz, Ewa Mijowska, Beata Zielinska**
Department of Nanomaterials Physicochemistry, Faculty of Chemical Technology and Engineering, West Pomeranian University of Technology in Szczecin, Piastow Ave. 42, 71-065 Szczecin, Poland
Corresponding author: *daria_baranowska@zut.edu.pl, **bzielinska@zut.edu.pl

Materials and methods

Duration optimization of fructose-assisted solvothermal modification of bulk graphitic carbon nitride

In the subsequent step, 40 mg of fructose (Sigma-Aldrich) was dissolved in a solution of 60 mL of distilled water and ethanol, mixed in a 1:1 volume ratio. Next, 400 mg of bulk-gCN was added to the prepared solution. The mixture was subjected to vigorous stirring for 0.5 h, followed by 0.5 h of sonication. Afterward, the resulting suspension was transferred into a 100 mL Teflon-lined autoclave, where it was maintained at a temperature of 180 °C for different durations (3, 6, 12, 18, or 24 h). After cooling down the suspension was centrifuged and washed three times with distilled water and ethanol, followed by drying at 60 °C overnight. The naming convention of the samples involved using “fructose_xh”, where “xh” indicates the duration of the reaction in the autoclave. For instance, “fructose_12h” signifies that bulk graphitic carbon nitride (bulk-gCN) was modified with fructose, and the reaction duration was 12 h.

Results and discussion

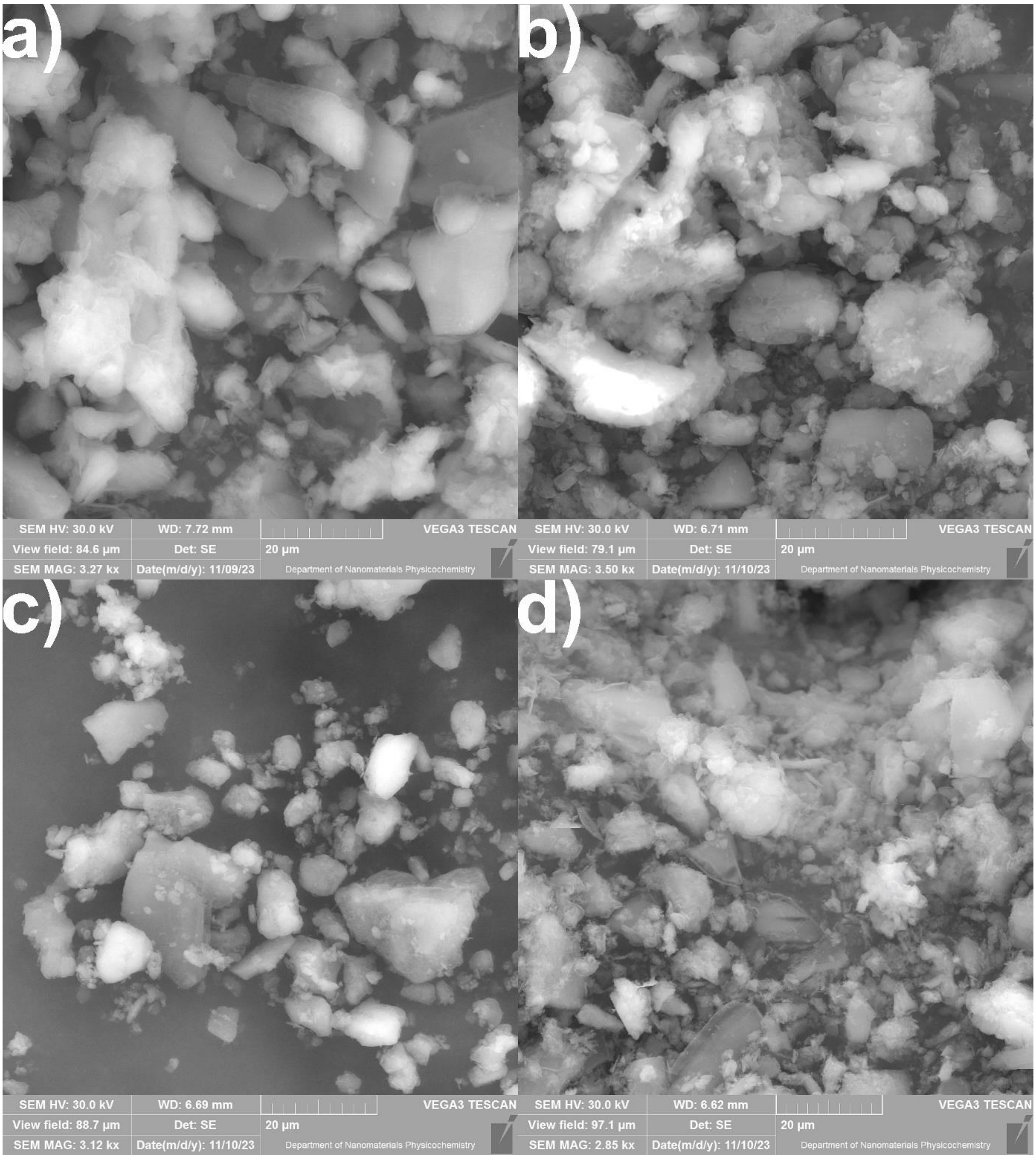

Fig. S1. SEM images of (a) bulk-gCN, (b) glucose_6h, (c) sucrose_6h, and (d) fructose_6h.

Table S1. AFM data of graphitic carbon nitride modified with different sugars.

Sample	Lateral size [nm]		Height [nm]		The mean number of layers
	Range	Mean	Range	Mean	
bulk-gCN	42-128	73.0	1.03-21.31	8.15	25
glucose_6h	23-132	48.1	1.07-19.49	5.22	16
sucrose_6h	39-147	75.1	1.89-21.73	8.25	25
fructose_6h	20-64	33.5	1.37-17.95	6.93	21

The XRD, and TGA results of the sugar-assisted solvothermal modification of graphitic carbon nitride *Fig. S2*. In detail, after sugar-assisted modification of graphitic carbon nitride, the peak at around 27° shifts towards higher angles, which indicates a concentration of the interlayer distance that has been correlated to the increased interaction induced by the more electronegative O-atoms replacing the C-atoms in the layer (oxygen doping) [S1, S2].

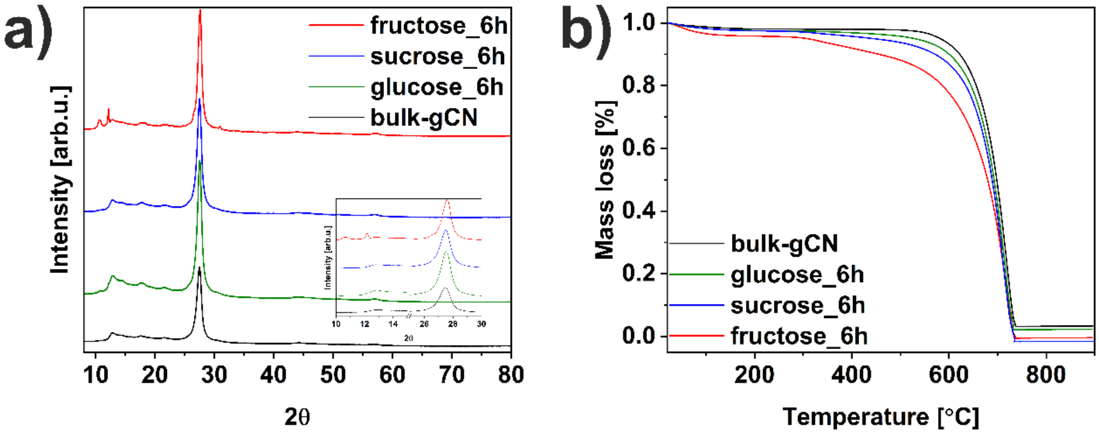

Fig. S2. (a) XRD diffractograms, (b) TGA spectra of graphitic carbon nitride modified with different sugars (glucose, sucrose, fructose).

The diffractograms of pure sugars (glucose, sucrose, and fructose) are presented in *Fig. S3a*. Glucose exhibits peaks at 10.49 (110), 11.97 (020), 14.77 (120), 17.21 (200), 18.88 (011), 19.84 (101), 20.73 (111), 23.20 (121), 24.86 (201), 25.55 (211), 28.59 (320), 34.70 (400), and 36.16° (151) according to card no. 00-024-1964. Sucrose shows peaks at 8.29 (100), 11.59 (001), 12.66 ($\bar{1}$01), 13.09 (110), 15.40 (011), 16.66 (200), 18.73 (111), 19.46 (210), 20.70 (020), 21.92 (120), 24.64 (211), 25.14 (300), 34.70 ($\bar{1}$03), and 47.54° (033) in accordance with card no. 00-024-1977. Fructose displays peaks at 13.18 (110), 14.21 (011), 14.71 (101), 17.20 (111), 19.49 (200), 20.17 (120), 20.86 (021), 21.03 (210), 22.12 (201), 24.09 (102), 25.85 (112), 28.46 (130), 31.44 (301), 32.61 (311), 34.61 (222), 35.09 (231), 35.98 (113), 39.24 (123), 40.15 (232), and 44.87° (303), which correspond to card no. 00-039-1839.

Fig. S3b displays the TGA/DTA results of pure sugars (glucose, sucrose, and fructose). The weight loss for fructose commenced at 130 °C, while glucose exhibited weight loss at 161 °C, and sucrose started losing weight at 194 °C [S3]. Furthermore, the melting points of fructose, glucose, and sucrose are 103, 148, and 179 °C, respectively [S3, S4]. To effectively modify graphitic carbon nitride using various sugars, it is advisable to maintain the reaction temperature above the respective melting points of utilized sugars.

The FTIR-ATR spectra of pure sugars (glucose, sucrose, and fructose) are depicted in *Fig. S3c*. All samples display similar absorption patterns, indicating a resemblance in the chemical structures of the green reducing agents used for the exfoliation of graphitic carbon nitride purpose. To elaborate, in the region of 1000 to 1200, the spectra exhibit vibration modes associated with C-C and C-O bonds, typical for carbohydrates [S5]. The region from 1350 to 1500 cm^{-1} shows the combination bands of C-O-C and C-O-H deformations, while absorption bands around 2900 cm^{-1} correspond to the aliphatic C-H stretching, and the absorption bands in the range of 3000-3600 cm^{-1} are attributed to the O-H stretching [S5]. Notably, the spectra of sucrose reveal overlapping absorption peaks primarily in the 3000-3600 cm^{-1} range, originating from both glucose and fructose. This is due to the fact that sucrose, known as disaccharide, is composed of these two monosaccharides.

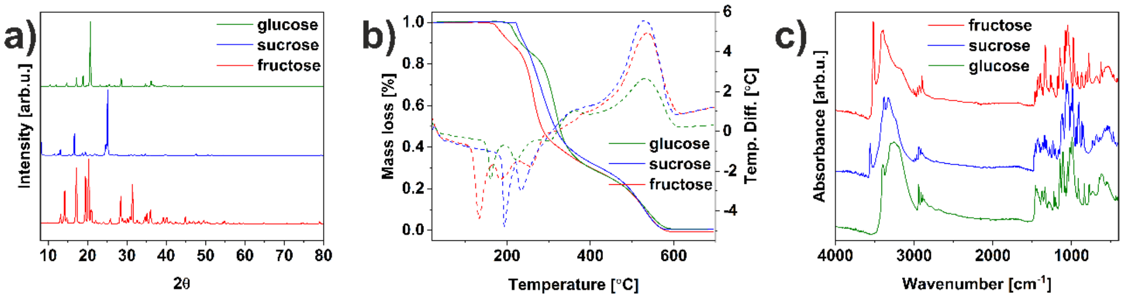

Fig. S3. (a) XRD diffractograms, (b) TGA spectra with corresponding DTA, (c) FTIR-ATR spectra of pristine glucose, sucrose, and fructose.

The XRD, FTIR-ATR, UV-vis with corresponding Tauc plot, PL, CA, and EIS results of the fructose-assisted solvothermal modification of graphitic carbon nitride for optimization of duration are depicted in *Fig. S4*. The description of graphitic carbon nitride is fully described in the main manuscript, thus in *Supplementary Material,* the authors described the differences of fructose-assisted modification of graphitic carbon nitride – duration of reaction dependence. In detail, the shift in peak position at around 27° (*Fig. S4a*) is influenced by the duration of the solvothermal reaction of fructose-assisted modification. This peak gradually shifts towards higher angles until 12 hours of reaction. Further extension of the solvothermal duration of the reaction (fructose_18h and fructose_24h) results in a shift in the opposite direction, indicating an improved interlayer stacking order [S2]. The absorption spectra of graphitic carbon nitride modified with fructose (*Fig. S4b*) reveal similar absorption peaks as observed in pristine bulk-gCN, indicating that the primary chemical structure of the graphitic carbon nitride structure remains intact, which aligns with XRD results. The energy band gaps (*Fig. S4cd*) were determined as 2.75, 2.80, 2.89, 2.84, 2.83, and 2.80 eV for bulk-gCN, fructose_3h, fructose_6h, fructose_12h, fructose_18h, and fructose_24h, respectively. Interestingly, the PL spectra (*Fig. S4e*) show that the duration of solvothermal reaction between 6-24 h has no significant impact on the recombination process. Both CA and EIS (*Fig. 4fg*) confirm that fructose_6h and fructose_12h have the highest mobility of charge carriers, thus both samples have similarly high photoactivity toward hydrogen production.

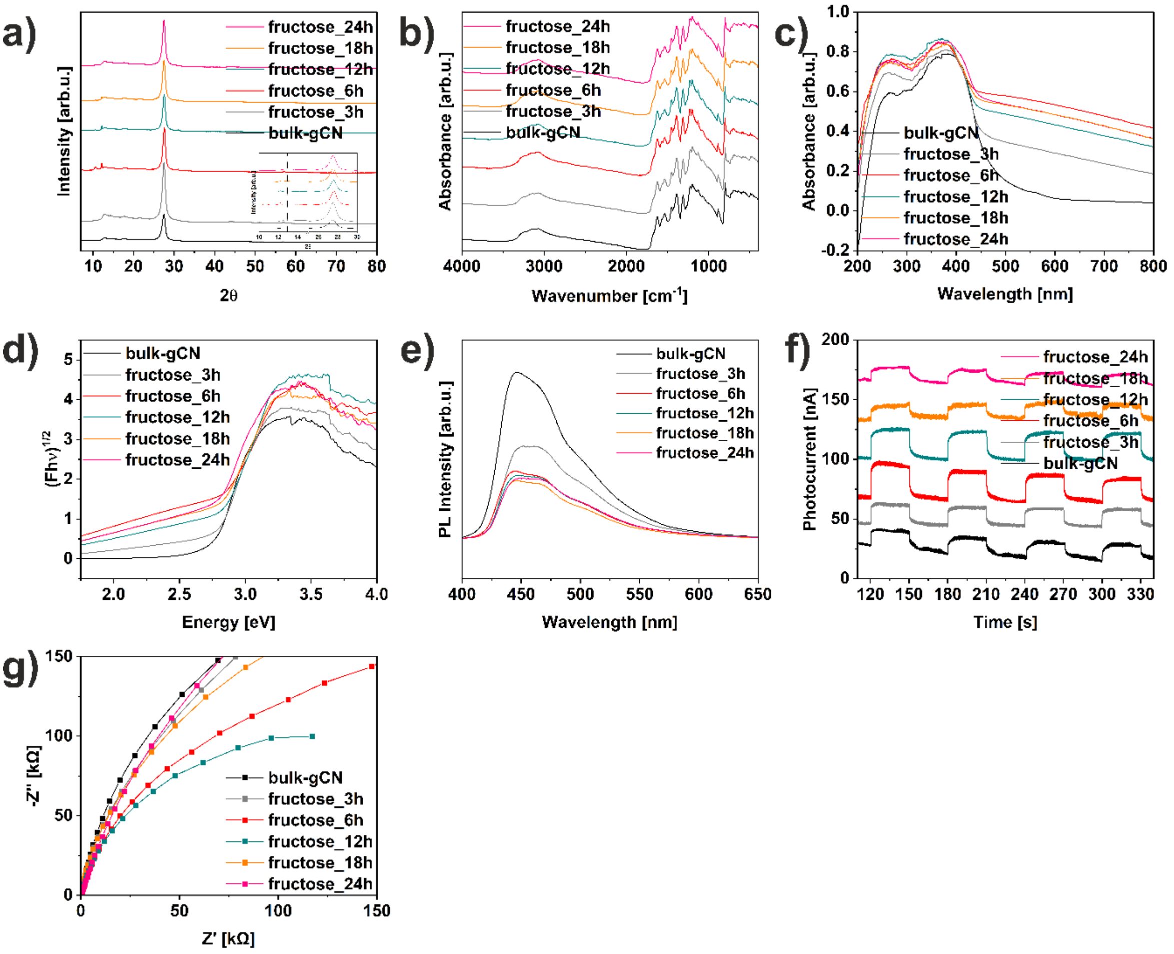

Fig. S4. (a) XRD diffractograms, (b)FTIR-ATR spectra, (c) DR/UV-vis spectra, (d) Tauc plot, (e) Photoluminescence emission spectra, (f) Chronoamperometry, (g) Electrochemical Impedance Spectroscopy of graphitic carbon nitride modified with fructose – influence of the solvothermal process time.

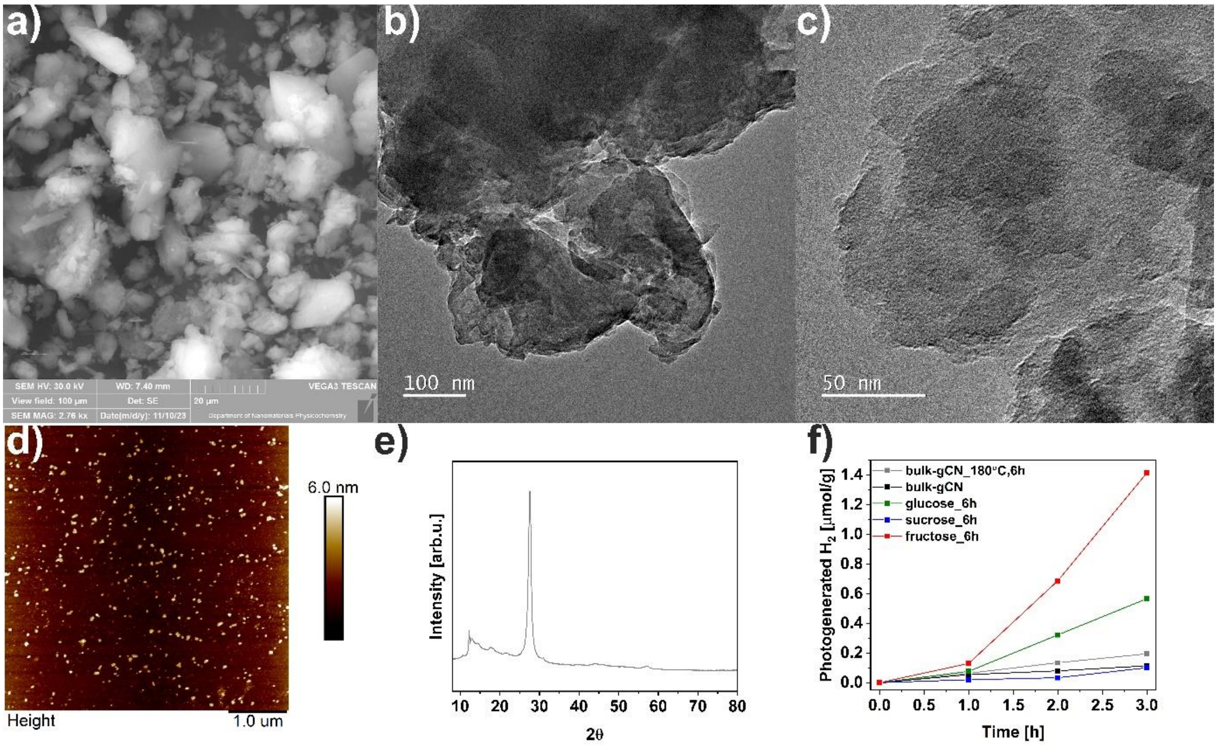

Fig. S5. (a) SEM image, (b, c) TEM images, (d) AFM image, (e) XRD diffractogram of bulk-gCN after solvothermal reaction in the absence of sugars, and (f) hydrogen evolution from water splitting of studied materials.

References

[S1] A. Speltini, A. Scalabrini, F. Maraschi, M. Sturini, A. Pisanu, L. Malavasi, A. Profumo, Improved photocatalytic H2 production assisted by aqueous glucose biomass by oxidized g-C3N4, International Journal of Hydrogen Energy, 43, 32, 2018, 14925-14933, https://doi.org/10.1016/j.ijhydene.2018.06.103.

[S2] D. Baranowska, K. Zielinkiewicz, T. Kedzierski, E. Mijowska, B. Zielinska, Heterostructure based on exfoliated graphitic carbon nitride coated by porous carbon for photocatalytic H2 evolution, International Journal of Hydrogen Energy, 47, 84, 2022, 35666-35679, https://doi.org/10.1016/j.ijhydene.2022.08.151.

[S3] R. Zhao, Z. Chang, Q. Jin, W. Li, B. Dong, X. Miao, Heterogeneous base catalytic transesterification synthesis of sucrose ester and parallel reaction control, Int. J. Food Sci. Technol., 2014, 49, 854-860, https://doi.org/10.1111/ijfs.12376.

[S4] M. Hurtta, I. Pitkanen, J. Knuutinen, Melting behaviour of d-sucrose, d-glucose and d-fructose, Carbohydrate Research, 2004, 339, 13, 2267-2273, https://doi.org/10.1016/j.carres.2004.06.022.

[S5] S. Sedaghat, E. Arshadi, P. Afshar, A. Nafar, R. Dabbagh, Rapid green biosynthesis and characterization of silver nanoparticles using glucose as a green route, Revue Roumaine de Chimie, 2019, 64, 5, 409-413, http://dx.doi.org/10.33224/rrch/2019.64.5.04.
